# Camera–GPS Sensor Fusion for Kinematic Characterization, Microsimulation Validation, and Macroscopic Capacity Modeling of Traffic-Calming Corridors

**DOI:** 10.3390/s26175340

**Published:** 2026-08-24

**Authors:** Deo Chimba, Wittness Mariki, Sunam Shrestha, Afia Yeboah

**Affiliations:** Department of Civil and Architectural Engineering, Tennessee State University, Nashville, TN 37209, USA; wmariki@my.tnstate.edu (W.M.); sshrest4@my.tnstate.edu (S.S.); ayeboah2@my.tnstate.edu (A.Y.)

**Keywords:** traffic calming, sensor fusion, GPS trajectory data, vehicle kinematics, microsimulation calibration, VISSIM, May–Keller model, speed hump, raised crosswalk, intelligent transportation systems

## Abstract

This study presents a sensor-fused field investigation and simulation-based analysis of four horizontal and vertical traffic-calming devices—two raised speed tables, a speed hump, and a raised crosswalk—installed along a 5250-ft two-lane residential collector in Nashville, TN, USA. A dual-sensor architecture combining a Miovision Scout video-based vehicle counter and WAAS/EGNOS-augmented GPS probe-vehicle logging (5 m 3-D RMS horizontal accuracy, 1 Hz sampling) was used to reconstruct 30 quality-controlled free-flow vehicle trajectories and 12-h per-lane volume counts. A spatial kinematic transform (a = v·dv/dx) was applied to extract device-specific approach-deceleration and post-device recovery-acceleration rates, and a three-parameter log-logistic cumulative-distribution function was fitted to the field-observed desired-speed percentiles (root-mean-square error below 0.043 for both speed-table devices). The camera- and GPS-derived observations were used to calibrate and statistically validate a PTV VISSIM microsimulation replica of the corridor, achieving a mean-speed calibration error of 0.71% or better at every device, a GEH statistic below 1.5 at all four analysis turning movements, and independent travel-time validation errors of 5.7–12.1%, within the accepted 15% threshold. The validated model was then used to reconstruct device- and spacing-specific May–Keller macroscopic speed–density–flow relationships, calibrated against simulated capacities of 650–775 vehicles per hour per lane at 350-, 700-, and 1050-ft device spacing. Results show capacity reductions of 20–33% relative to free-flow conditions and yield kinematically derived maximum recommended spacings of 265–630 ft to maintain crossing speeds at or below 15 mph, depending on device geometry. The findings demonstrate a reproducible, low-cost sensor-fusion workflow for quantifying the safety–capacity trade-off of traffic-calming corridors and for informing the design of sensor-in-the-loop adaptive-calming infrastructure.

## 1. Introduction

Traffic-calming devices—speed humps, speed tables, raised crosswalks, chicanes, and related vertical or horizontal deflections—are among the most widely deployed low-cost countermeasures for reducing operating speeds and improving pedestrian safety on urban and residential collectors [1,2,3,4,5]. Their safety benefit, however, is inseparably coupled to an operational cost: every forced deceleration–recovery cycle removes kinetic energy from the traffic stream, and when devices are installed too close together, the recovery phase of one device overlaps the approach phase of the next, compounding a capacity penalty that is rarely quantified with the same rigor as the safety benefit [6,7,8]. Historically, traffic-calming evaluations have relied on manual radar-gun speed sampling, pneumatic tube counts, or small, underpowered instrumented-vehicle fleets [9,10,11,12]. Each method captures only a partial slice of the vehicle population and cannot resolve continuous, distance-referenced speed trajectories through the influence zone of a device. The emergence of low-cost video-based vehicle sensors (e.g., Miovision Scout) capable of per-lane, per-movement 12-h classification counts [13], combined with consumer-to-survey-grade Global Positioning System (GPS) receivers offering sub-5-m horizontal accuracy and 1 Hz kinematic sampling [14], now allows a single field deployment to simultaneously capture (i) aggregate volume and turning-movement demand and (ii) continuous, vehicle-level speed–position trajectories through a corridor. This sensor pairing—a fixed camera node and mobile GPS probe vehicles—is a canonical instance of the camera–GPS sensor-fusion architectures increasingly used across intelligent transportation systems (ITS) applications, from autonomous-vehicle perception [15,16] to macroscopic speed and travel-time reconstruction [17,18].

Despite this instrumentation maturity, the traffic-calming literature has not fully exploited sensor-fused field data to (i) parametrize device-specific kinematic models of the deceleration–recovery envelope, (ii) statistically validate microsimulation replicas of the corridor against independently observed GPS trajectories and camera counts, and (iii) propagate the validated microscopic behavior into macroscopic capacity and minimum-spacing design guidance. Prior microsimulation studies of device spacing and capacity [7,8] typically assume generic car-following parameters rather than field-calibrated ones, while pure before/after speed studies [4,5,19] rarely close the loop into a capacity or spacing recommendation.

This study is organized around four research questions (RQ1–RQ4). RQ1: How can fixed-camera volume counts and continuous GPS probe-vehicle trajectories be fused into a single field protocol that captures both corridor-wide exposure and device-level driver response? RQ2: What are the distinct approach-deceleration and recovery-acceleration kinematic signatures of different traffic-calming device geometries (speed table, speed hump, raised crosswalk)? RQ3: Can a microsimulation replica calibrated directly to fused sensor trajectories achieve materially better speed and volume fidelity than one calibrated only to generic car-following defaults, as in prior device-spacing studies [7,8]? RQ4: How does device spacing propagate through microscopic driver behavior into macroscopic corridor capacity, and what spacing is required to keep downstream crossing speeds within a pedestrian-safety threshold?

This paper addresses that gap using a four-device residential corridor case study—two raised speed tables (17-ft and 21-ft top width), a speed hump, and a raised crosswalk—instrumented with a fused Miovision camera and GPS probe-vehicle sensor network. The specific contributions of this work are:

1. A field-validated camera–GPS sensor-fusion protocol for simultaneous volume and continuous trajectory data collection at multiple traffic-calming devices along a single corridor.
2. A distance-referenced kinematic feature-extraction model (spatial kinematic transform) that decomposes each GPS-observed trajectory into an approach-deceleration zone, a minimum crossing speed and position, and a post-device recovery-acceleration zone, without requiring assumptions about constant time-step sampling.
3. A parametric log-logistic desired-speed distribution fitted directly to the field percentile data, providing a compact, continuously differentiable representation of the free-flow speed distribution for simulation seeding or design-speed selection.
4. A field-calibrated and multi-metric-validated (mean-speed error, GEH statistic, travel-time error) PTV VISSIM microsimulation replica of the corridor.
5. A device- and spacing-specific calibration of the generalized May–Keller macroscopic speed–density model, anchored to VISSIM-derived capacities at three device spacings (350, 700, and 1050 ft), from which speed–flow–density diagrams and capacity-reduction percentages are reconstructed.
6. Kinematically derived, device-specific maximum spacing recommendations for maintaining crossing speeds at or below a 15-mph pedestrian-safety threshold.

## 2. Related Work

### 2.1. Effectiveness and Trade-Offs of Traffic-Calming Devices

Traffic-calming techniques—physical deflections (speed humps, tables, raised crosswalks), horizontal deflections (chicanes, curb extensions), and visual or psychological treatments—have been studied since at least the early 1990s [1,2,3]. Meta-analyses and state-of-the-practice reviews consistently report 15–40% reductions in the 85th-percentile operating speed within a device’s influence zone [2,4,5,11], but with a well-documented decay of effect with distance from the device and a partial or full recovery of speed between closely spaced devices [6,19]. Ewing’s widely cited review [4] and the subsequent update by Ewing and Kooshian [5] establish speed and volume reduction as the primary metrics of calming effectiveness, while more recent systematic reviews call for quantitative evaluation frameworks that couple field measurement with simulation-based extrapolation [20]. Environmental and driver-behavior consequences of calming—increased emissions from repeated acceleration cycles [21,22], perceived-speed shifts on approach to devices [23], and behavioral adaptation measured in driving simulators [19,24]—further motivate a kinematically grounded, rather than purely before/after, characterization of the deceleration–recovery cycle.

### 2.2. Microsimulation and Capacity Impacts of Device Spacing

The operational, as opposed to safety, cost of traffic calming has received comparatively less field-validated attention. García et al. [7] and Shirmohammadi et al. [8] used VISSIM microsimulation to show that closer device spacing produces measurable capacity reductions, but calibrated their car-following parameters to generic literature defaults rather than to site-specific GPS or camera data. Lee et al. [6] proposed an evaluation framework combining speed, volume, and safety indices but did not extend it into a macroscopic capacity model. The Highway Capacity Manual [25] and classical traffic-engineering texts [26] establish the GEH statistic and percent-difference thresholds for simulation validation that this study adopts directly.

### 2.3. Sensor Fusion in Intelligent Transportation Systems

Camera- and GPS-based sensing have each independently matured as low-cost alternatives to inductive loops and radar for volume and speed data collection. Video-analytics platforms such as Miovision Scout [13] provide automated, per-lane, per-class 12-h counts without pavement-invasive installation. Survey-grade WAAS/EGNOS-augmented GPS receivers achieve sub-5-m 3-D RMS horizontal accuracy and 0.1 m/s velocity accuracy at 1 Hz [14], sufficient to resolve second-by-second deceleration profiles through a device’s influence zone. The combination of fixed-camera and mobile-GPS sensing is a specific case of the broader sensor-fusion paradigm now standard in ITS and autonomous-vehicle perception research: recent Sensors-journal work demonstrates camera–radar and camera–LiDAR fusion for vehicle detection and hazard resolution [15,16], multi-sensor architectures for perception in complex traffic scenes [16], and systematic reviews of data-fusion techniques across the ITS stack [17]. GPS trajectory mining specifically has been used for stop/go classification [27], macroscopic speed and travel-time reconstruction via the two-fluid model [28], and vehicle classification from onboard inertial signals [29]. Deep-learning trajectory models (CNN–LSTM) have recently been applied to conflict prediction on curved alignments [30], illustrating the growing role of machine learning atop fused sensor streams. Emerging smart traffic-calming concepts—adaptive speed humps and bumps that sense approaching vehicles and modulate their profile in real time [31,32]—represent a natural extension of the present sensor-fusion framework toward closed-loop, sensor-actuated calming infrastructure, an application area explicitly within the scope of the Sensors journal.

### 2.4. Macroscopic Traffic-Flow Theory

Classical single-regime macroscopic speed–density models—Greenshields’ linear model, Underwood’s exponential model [33], and the generalized non-linear May–Keller family [34,35,36]—remain the standard tool for translating microscopic (vehicle-level) behavior into macroscopic capacity and level-of-service estimates [25]. The May–Keller model, in particular, nests Greenshields’ and Underwood’s models as special cases through its shape parameters and is well suited to representing the compressed, capacity-constrained flow regime induced by closely spaced traffic-calming devices [34,35]. This study calibrates the May–Keller model directly against VISSIM-simulated device- and spacing-specific capacities rather than assuming a single generic parameter set for the whole corridor—an approach that, to the authors’ knowledge, has not previously been applied to traffic-calming capacity analysis using sensor-fused field data as the calibration target.

### 2.5. Novelty Relative to Prior Work

Table 1 situates the present study’s methodological contribution against the traffic-calming evaluation and microsimulation literature discussed above, across the five dimensions in which this study departs from prior work.

## 3. Materials and Methods

### 3.1. Study Site

The study corridor is Oakhill Valley Lane, a two-lane residential collector street in Nashville, TN, USA, extending approximately 5250 ft between two stop-controlled intersections (Robertson Road and Van Lee Drive to the north and Churchwood Drive to the south, with Oak Hill School situated between them). The corridor carries a posted speed limit of 30 mph, dropping to 15 mph in the immediate vicinity of each traffic-calming device. Four devices are installed along the corridor: two raised speed tables (17-ft and 21-ft top width, referred to hereafter as Speed Table 1 and Speed Table 2), one speed hump, and one raised crosswalk located near the school frontage. The corridor also includes on-street parking and an after-hours access gate near the school, both of which were represented in the microsimulation network coding.

This study follows a seven-stage analytical framework linking field sensing to device-spacing guidance, Table 2. The framework begins with field sensor deployment: a fixed Miovision Scout camera records twelve-hour per-lane traffic counts while ten drivers instrumented with a WAAS/EGNOS-enabled GPS receiver complete forty round trips through the corridor at 1 Hz. These raw trajectories then pass through data quality control, which removes non-free-flow, template-duplicated, and physically implausible runs, retaining thirty validated trajectories. Kinematic feature extraction and desired-speed-distribution estimation derive the spatial acceleration profile a(x) = v·dv/dx (Equations (1) and (2)) from the validated trajectories and fit a log–logistic cumulative distribution to the percentile speeds (Equation (3)). These field-derived speed and acceleration profiles anchor microsimulation calibration and validation, in which a PTV VISSIM (VISSIM 2020) Wiedemann-74 car-following model is calibrated to the GPS-observed mean speeds (Equation (4)), checked against field counts with the GEH statistic (Equation (5)), sized for an adequate number of replications (Equation (6)), and validated independently against measured travel times. The calibrated microsimulation supplies device- and spacing-specific capacity estimates for the macroscopic capacity model, based on the May–Keller speed–density–flow relationship (Equations (7) and (8)). Finally, the kinematic and capacity results are combined in the spacing and design recommendations: a theoretical minimum device spacing derived from deceleration/reacceleration kinematics (Equation (9)), a maximum spacing tied to a 15-mph design-speed threshold, and statistical significance testing (ANOVA/Fisher LSD) across device types. These results are then translated into device-specific design guidance and discuss extensibility to real-time, sensor-actuated calming devices.

### 3.2. Sensor Architecture

**Figure 1:**
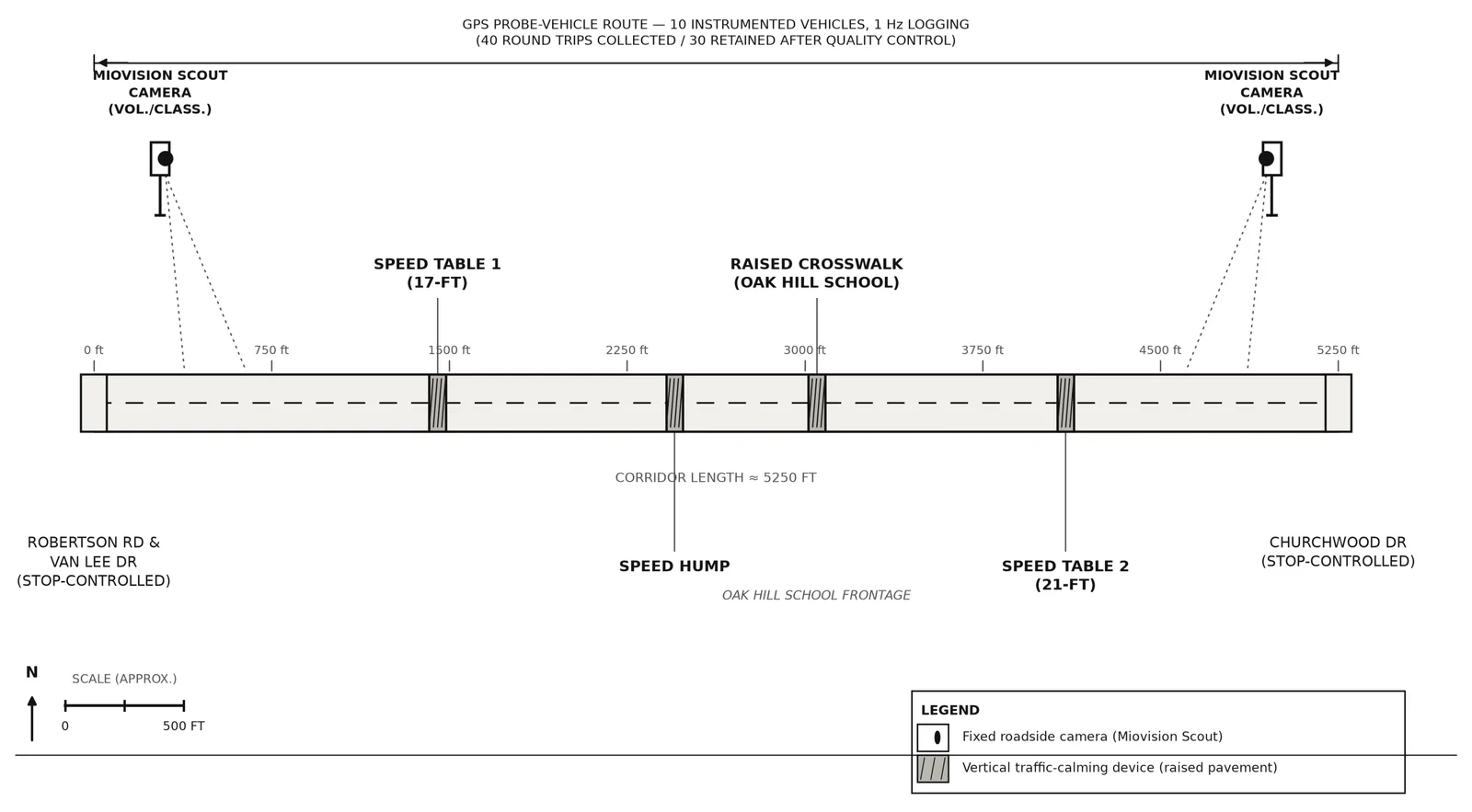
Oakhill Valley Lane corridor: fixed camera and traffic-calming device placement, with GPS probe-vehicle route (schematic, not to exact scale).

Two complementary sensing modalities were fused to characterize corridor operations. Fixed video sensor—volume and classification, Figure 1. A Miovision Scout portable video-based traffic sensor [13] was deployed at each corridor approach to record continuous 12-h weekday counts (07:00–18:00), post-processed into per-lane, per-vehicle-class turning-movement volumes at each of the four analysis segments used for microsimulation validation. Mobile GPS probe vehicles—continuous trajectories. Ten instrumented probe vehicles, each carrying a WAAS/EGNOS-augmented GPS receiver (5 m 3-D root-mean-square horizontal accuracy, 0.1 m/s velocity accuracy, 1 µs time-synchronization accuracy, 1 Hz logging rate) [14], completed repeated round trips along the corridor. A total of 40 round-trip logs were collected; 10 were excluded due to non-free-flow conditions (queuing behind a lead vehicle), pedestrian interference, transient signal loss, or physically implausible acceleration spikes, leaving 30 validated free-flow runs retained for percentile and calibration analysis. The fusion of the two sensor streams follows a common spatial reference frame: camera-derived volumes are tied to the same four corridor segments used for GEH-based volume validation, while GPS trajectories are tied to a continuous distance coordinate measured from each device’s centerline (negative values upstream, positive downstream), enabling direct overlay of speed-versus-position profiles across vehicles and across devices.

### 3.3. Data Quality Control

Trajectory logs exported from the field GPS receivers and from the calibrated VISSIM replica were audited row-by-row prior to kinematic analysis. Two data-quality issues were identified and are disclosed here for full transparency. First, several exported trajectory columns were found to contain identical, template-duplicated values across all four device worksheets—an artifact of the spreadsheet export process rather than genuine device-specific telemetry—and were excluded from any per-device analysis. Second, after isolating the genuinely device-differentiated position–speed columns, usable continuous ranges were found to vary in length and completeness by device: Speed Table 1 retained a full 32-point clean profile (−49.2 ft to +727.1 ft), Speed Table 2 retained 19 points (−49.2 ft to +748.2 ft), the speed hump retained 13 points but exhibited a non-physical, non-unimodal speed oscillation inconsistent with a single approach–crossing–recovery event and was therefore excluded from trajectory-level kinematic and figure reporting (its validated aggregate statistics from the original field study were retained), and the raised crosswalk retained seven points spanning only the crossing and early-recovery zone (469.5–773.7 ft), without a full upstream approach segment. All device-level aggregate statistics (percentiles, means, standard deviations, calibration and validation metrics, and capacity values) used in this study were taken directly and without modification from the field study’s validated report tables; only the row-level trajectory reconstruction in (Figure 1) is limited to the three devices with physically consistent clean data.

### 3.4. Kinematic Feature-Extraction Model

For each of the three devices with clean position-referenced trajectories, a distance-referenced (rather than time-referenced) kinematic model was used, since GPS logs are unevenly spaced in time but are directly indexed by position along the corridor. Given paired samples of position x and speed v, the instantaneous acceleration a(x) is obtained through the spatial kinematic identity [26]:

(1) $$a\left(x\right)=\nu \left(x\right)\;\cdot \;d\nu /dx$$

which follows directly from the chain rule a = dv/dt = (dv/dx)(dx/dt) = v·(dv/dx), and is exact regardless of the possibly irregular time spacing between GPS fixes. The position of minimum crossing speed, *x_min_*, was located from the raw trajectory using a heavily smoothed cubic spline (smoothing factor scaled to sample count) to suppress point-level GPS/simulation export noise while preserving the single-minimum shape of the approach–crossing–recovery envelope. The approach-zone-average deceleration and recovery-zone-average acceleration were then computed from the constant-acceleration kinematic identity [26,34]:

(2) $$a_{zone}=\left({V_{end}}^{2}-{V_{min}}^{2}\right)/\left(2\cdot \Delta x\right)$$

applied separately to the approach zone (Δx = *x_min_* − *x_start_*, *v_end_* = *v_start_*) and the recovery zone (Δx = *x_end_* − *x_min_*, *v_end_* = *v_end_*), where *v_min_* is the spline-estimated minimum crossing speed. This zone-averaged formulation—the same underlying kinematic identity used in Equation (9) below for minimum-spacing design—is more robust to point-level trajectory noise than a purely local derivative estimate and yields a single physically interpretable rate per zone, directly comparable across devices and against the field study’s own reported average deceleration values.

### 3.5. Desired-Speed Distribution Model

The field study’s percentile-based desired-speed tables (Table 2) were converted to individual driver desired-speed estimates using the linear percentile-interpolation formula, consistent with standard speed-study percentile estimation practice [25]:

*S_D_* = [(*P_D_* − *P_min_*)/(*P_max_* − *P_min_*)]·(*S_max_* − *S_min_*) + *S_min_* (3)

where *P_D_* is a driver’s assigned percentile rank and *S_min_*, *S_max_* are the minimum and maximum observed percentile speeds. To obtain a continuously differentiable, parametric representation of the desired-speed distribution suitable for simulation seeding, a three-parameter log–logistic cumulative distribution function was fit via non-linear least squares to the seven field-observed percentile pairs (first, seventh, fifteenth, fiftieth, eighty-fifth, ninety-fifth, and one-hundredth percentile) reported for each speed-table device, minimizing the root-mean-square error between fitted and observed cumulative percentiles. The speed hump and raised crosswalk were not included in this fit because the underlying field study tabulated a full seven-point percentile distribution only for the two speed-table devices (Table 2).

### 3.6. Microsimulation Calibration and Validation Framework

The corridor, including all four calming devices, both controlling intersections, on-street parking, and the after-hours access gate, was coded in PTV VISSIM [37], with Wiedemann 74 car-following parameters seeded initially from published defaults [38] and then adjusted so that simulated mean speeds at each device matched the GPS-derived observed means within calibration tolerance. Calibration accuracy was assessed via percent difference between observed and simulated mean crossing speed,

%Diff = |*S_obs_* − *S_sim_*|/*S_obs_* × 100 (4)

Volume and turning-movement validation used the GEH statistic,

GEH = √[2(*m* − *c*)^2^/(*m* + *c*)] (5)

where m and c are the camera-observed and simulated hourly volumes, respectively, at each of the four analysis turning movements; a GEH below five is the accepted Highway Capacity Manual/FHWA threshold for an individual movement [25,39]. Independent travel-time validation used 10 additional GPS round-trip runs per direction (northbound and southbound) not used in calibration, with acceptance requiring a percent travel-time error below 15% per FHWA guidance [39]. The number of simulation replications required for a statistically stable mean output was determined from the standard confidence-interval sample-size formula [40]:

C = 2·*t*_(1−α/2, N−1)_·(*s*/√*N*) (6)

evaluated at a 95% confidence level, which indicated a minimum of 10 replications; all reported simulated values are means across the retained replications.

### 3.7. Macroscopic Capacity Model

The generalized non-linear speed–density relationship of May and Keller [34], a two-parameter generalization that nests the Greenshields [41] and Underwood [33] models, was used to reconstruct device- and spacing-specific macroscopic flow diagrams:

*U*(*K*) = [*U_f_*^1−*m*^ + *c*·*K*^*l*−1^]^1/(1−*m*)^ (7)

*Q*(*K*) = *K*·*U*(*K*) (8)

where *U_f_* = 30 mph is the free-flow speed (matching the corridor’s posted limit and VISSIM base-case calibration), K is traffic density in vehicles per mile per lane (veh/mi/ln), and m, l, c are shape and scale parameters. Because the field study did not measure density directly, the model was calibrated using two physically grounded boundary conditions rather than a free four-parameter fit: (i) the jam-density condition U(*K_j_*) = 0 at *K_j_* = 190 veh/mi/ln (a standard passenger-car jam density [25]) pins c as a function of m for a fixed-density exponent l = 2 (the quadratic case), and (ii) the resulting single free parameter m is then solved, for each device and each of the three simulated device spacings (350, 700, and 1050 ft), such that the resulting curve’s maximum flow over all K exactly reproduces VISSIM’s simulated capacity at that spacing. Because the capacity target in the second boundary condition is taken directly from the device- and spacing-specific VISSIM simulation rather than from Kj itself, the reconstructed capacity value at each device/spacing combination is insensitive to the specific jam-density value assumed; Kj instead governs only the curvature of the reconstructed speed–density relationship away from the capacity point, an effect explicitly flagged as a model-derived (not independently field-validated) limitation in Section 6. This anchoring approach ensures that every reconstructed speed–flow–density diagram passes through a capacity point grounded in the study’s own simulated data, rather than an assumed generic shape parameter.

### 3.8. Minimum-Spacing and Recommended-Spacing Model

The same constant-deceleration kinematic identity underlying Equation (2) was used, in the field study’s original design context, to derive the theoretical minimum spacing between two devices such that a vehicle decelerating to a device crossing speed could reaccelerate back to the posted design speed before reaching the next device [26]:

(9) $$S_{\min}=\left(V^{2}-{V_{0}}^{2}\right)/\left(2a\right)$$

with V0 = 6.92 mph (approach crossing speed), V = 30 mph (design return speed), and a = 2.5 m/s^2^ (comfortable passenger-vehicle acceleration), yielding a theoretical minimum spacing of 111.65 ft; a practical spacing of 350 ft was adopted as the shortest simulated scenario to remain conservative relative to this theoretical minimum. The complementary, safety-oriented recommended maximum spacing values were derived from VISSIM-simulated crossing-speed profiles at intermediate spacings, selecting the largest spacing at which the simulated 85th-percentile crossing speed at the downstream device remained at or below a 15-mph pedestrian-safety threshold.

### 3.9. Statistical Analysis

One-way analysis of variance (ANOVA) was used to test whether mean simulated capacity differed significantly across the three device spacings (350, 700, and 1050 ft), followed by Fisher’s least-significant-difference (LSD) post hoc pairwise comparison between the 350-ft and 700-ft spacing conditions.

## 4. Results

### 4.1. Field-Observed Desired-Speed Distribution

Table 3 reports the field-observed cumulative desired-speed percentiles for the two speed-table devices, the only devices for which the source field study tabulated a complete seven-point percentile distribution. Both devices show a similar overall spread (first-percentile speeds of 7.8–9.8 mph, one-hundredth-percentile speeds of 25.3–25.9 mph), with Speed Table 2 (21-ft) exhibiting a slightly higher median speed (14.2 mph versus 13.3 mph for Speed Table 1).

**Figure 2:**
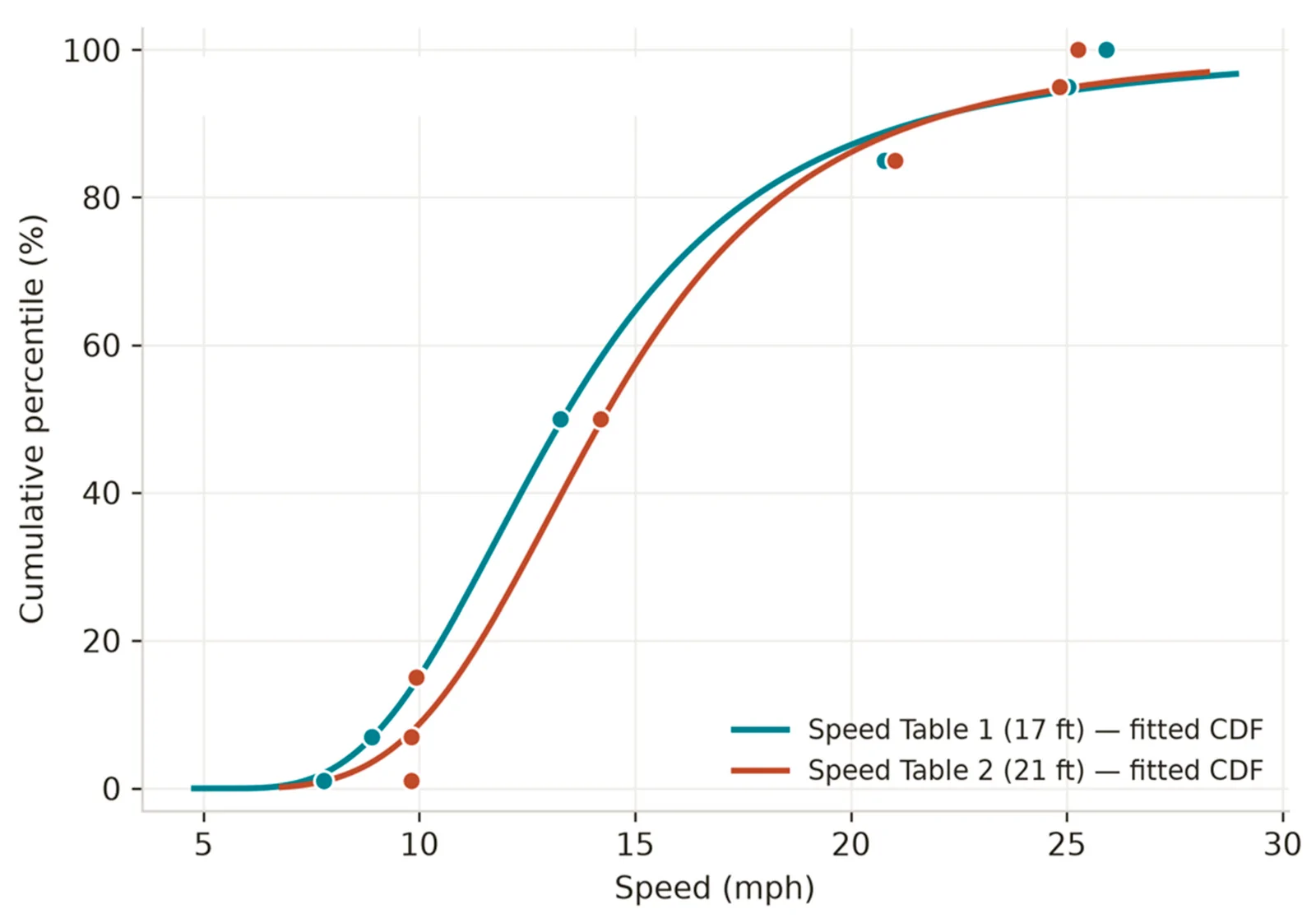
Field-observed desired-speed percentiles (markers) and fitted log–logistic cumulative distribution functions (curves) for Speed Table 1 (17 ft) and Speed Table 2 (21 ft).

A three-parameter log–logistic cumulative distribution function was fitted to each device’s percentile set. Table 4 reports the fitted parameters and root-mean-square error (RMSE) between the fitted and observed cumulative percentiles; both fits achieve an RMSE below 0.043 (i.e., well under five cumulative-percentage-points average deviation), confirming that a single continuous parametric family adequately represents the field-observed desired-speed distribution at both devices. Figure 2 overlays the fitted curves against the observed percentile markers.

### 4.2. Descriptive Speed and Deceleration Statistics

**Figure 3:**
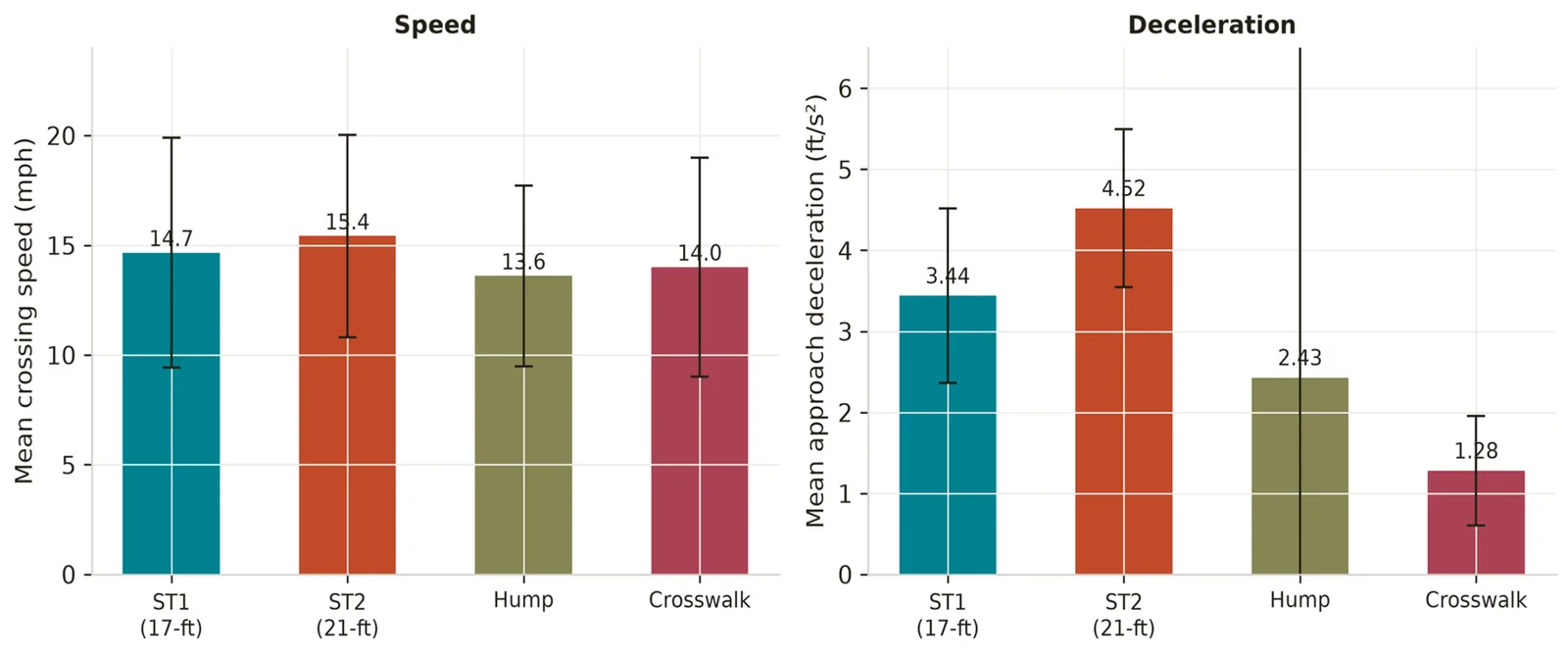
Mean crossing speed (**left**) and mean approach deceleration (**right**) by device, with error bars showing one standard deviation.

Table 5 and Figure 3 summarize the mean and standard deviation of crossing speed and approach deceleration across all four devices, taken from the field study’s validated descriptive statistics. Mean crossing speeds range from 13.6 mph (speed hump) to 15.4 mph (Speed Table 2, 21-ft), all substantially below the 30-mph posted corridor speed and consistent with the devices’ design intent. Mean approach deceleration is highest at Speed Table 2 (4.52 ft/s^2^) and lowest at the raised crosswalk (1.28 ft/s^2^), indicating that the wider 21-ft speed table—despite its longer top width—was associated with a sharper approach deceleration than the narrower 17-ft table, likely reflecting differences in upstream sight distance or driver expectancy rather than device geometry alone.

### 4.3. Kinematic Trajectory Reconstruction

**Figure 4:**
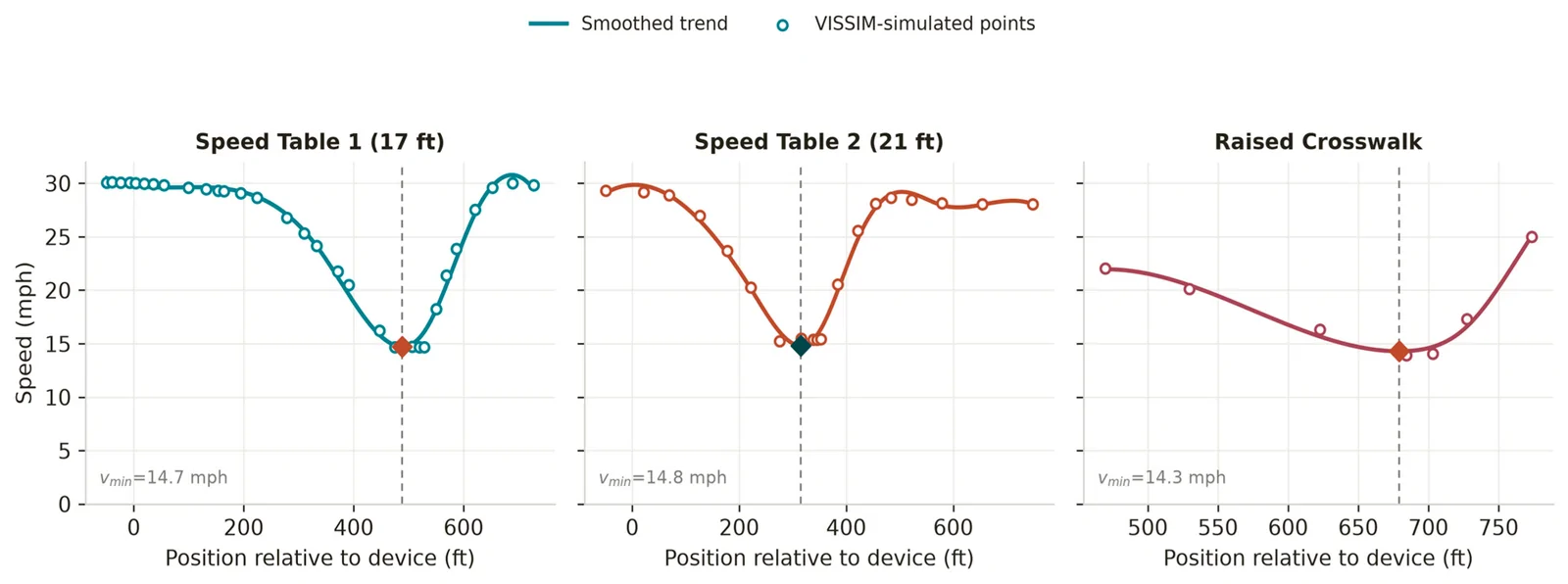
Reconstructed speed-versus-position trajectories at Speed Table 1, Speed Table 2, and the Raised Crosswalk. Raw GPS/simulation-derived samples (points), smoothed trend (line), and identified minimum crossing speed and position (diamond marker) are shown for each device.

Figure 4 presents the reconstructed speed-versus-position trajectories for the three devices with physically consistent clean GPS data: Speed Table 1, Speed Table 2, and the raised crosswalk. Each panel shows the raw device-referenced position–speed samples, a heavily smoothed trend curve used to locate the minimum-speed point, and a marker at the identified minimum crossing speed and position. Table 6 reports the resulting zone-average kinematic features computed from Equation (2).

Across the two speed tables, the approach-zone deceleration is markedly lower at Speed Table 1 (1.38 ft/s^2^ over a 537-ft zone) than the zone-average value implied at Speed Table 2 (1.89 ft/s^2^ over a 364-ft zone), i.e., the 21-ft table decelerates traffic over a shorter distance and at a higher zone-average rate than the 17-ft table, consistent with the higher point-estimate deceleration reported for Speed Table 2 in the aggregate statistics of Table 5. Recovery-zone acceleration shows the opposite pattern: Speed Table 1 recovers over a much shorter zone (239 ft, 3.02 ft/s^2^) than Speed Table 2 (434 ft, 1.41 ft/s^2^), suggesting that downstream geometry or driver behavior after the 17-ft table permits a more aggressive return to free-flow speed. The raised crosswalk trajectory, limited to only the crossing and early-recovery zone, shows the shortest recovery zone of all three devices (94.5 ft) but the highest recovery acceleration (4.80 ft/s^2^), which is physically plausible given its partial data coverage but should be interpreted cautiously given the small sample (n = 7 points).

### 4.4. Microsimulation Calibration and Validation

**Figure 5:**
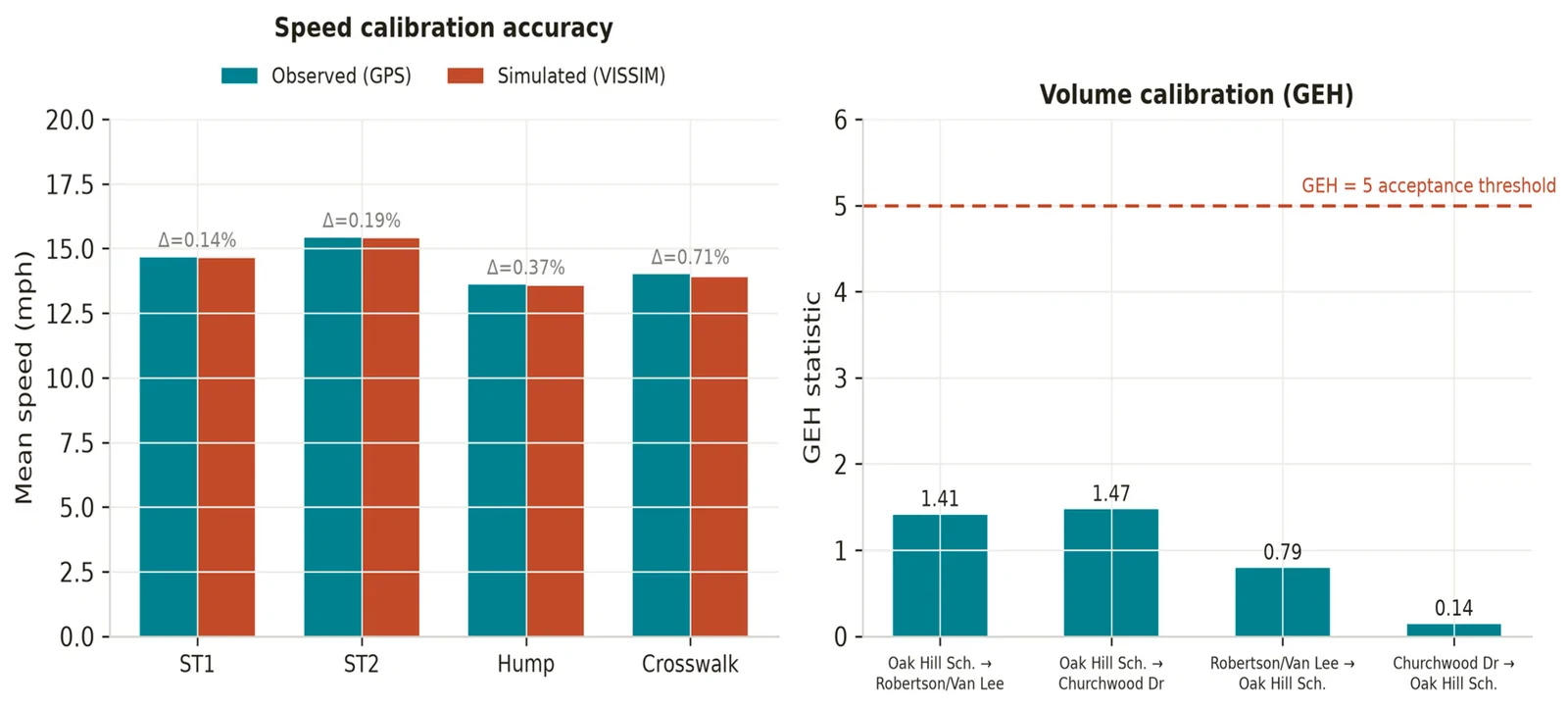
(**Left**): observed versus VISSIM-simulated mean crossing speed by device, with percent-difference labels (Equation (4)). (**Right**): GEH statistic by turning movement against the acceptance threshold of five (Equation (5)).

Speed calibration accuracy at all four devices is excellent: the percent difference between GPS-observed and VISSIM-simulated mean crossing speed (Equation (4)) ranges from 0.14% (Speed Table 1) to 0.71% (Raised Crosswalk), well within the 5–10% tolerance conventionally applied in microsimulation calibration [25,39] (Table 7). Volume validation at the four analysis turning movements likewise passes the GEH acceptance threshold of five at every movement, with GEH values ranging from 0.14 to 1.47 (Table 8). Independent travel-time validation, using GPS runs withheld from calibration, shows a 5.7% error northbound and a 12.1% error southbound (Table 9), both within the 15% FHWA acceptance threshold [39]; the larger southbound error. Figure 5 summarizes calibration and GEH results graphically.

### 4.5. Macroscopic Speed–Density–Flow Reconstruction

**Figure 6:**
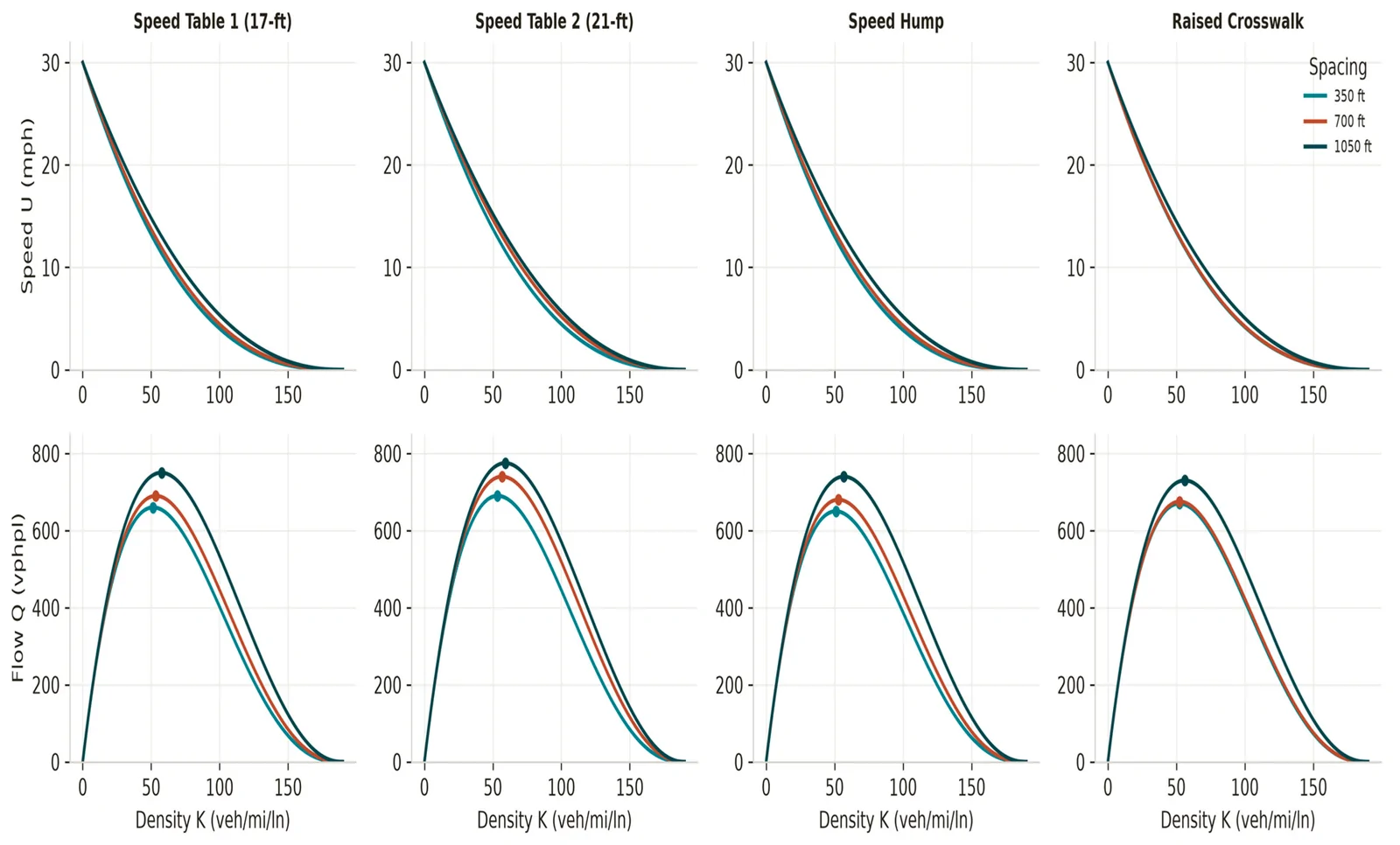
Reconstructed May–Keller speed–density (**top row**) and speed–flow (**bottom row**) diagrams for all four devices at 350-, 700-, and 1050-ft spacing.

The May–Keller model (Equations (7) and (8)), calibrated device-by-device and spacing-by-spacing against the VISSIM-simulated capacities expressed in vehicles per hour per lane (vphpl, Table 10), reproduces the target capacity exactly at every device/spacing combination (Table 11), with calibrated shape parameter m ranging narrowly from 0.550 to 0.635 across all twelve device–spacing combinations. This is a relatively tight range, suggesting that device geometry has a second-order effect on the underlying flow-model shape once capacity is anchored, while spacing itself, through its direct effect on achievable capacity, remains the dominant explanatory variable. Figure 6 presents the full set of reconstructed speed–density and speed–flow diagrams for all four devices at all three simulated spacings.

### 4.6. Capacity Reduction and Recommended Device Spacing

**Figure 7:**
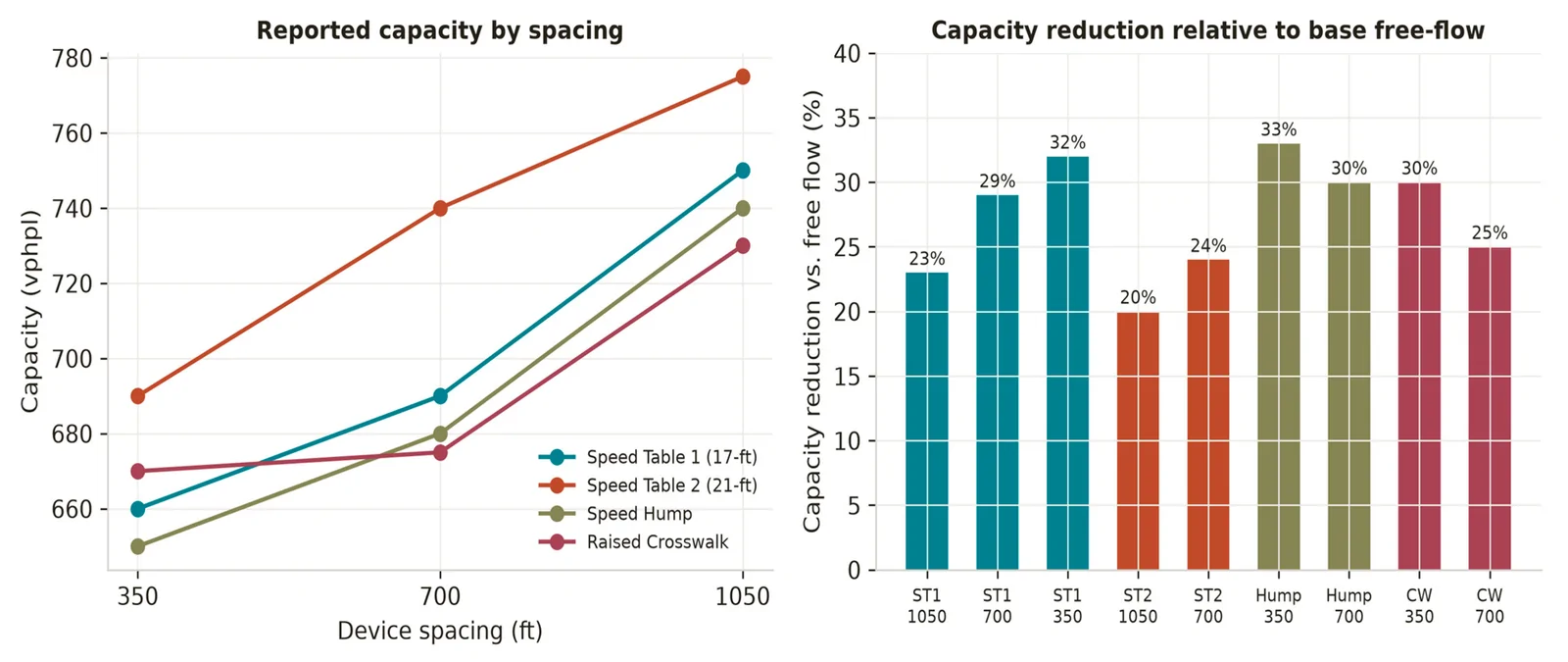
(**Left**): simulated capacity versus device spacing by device. (**Right**): capacity reduction (%) relative to free-flow base capacity, by device and spacing.

**Figure 8:**
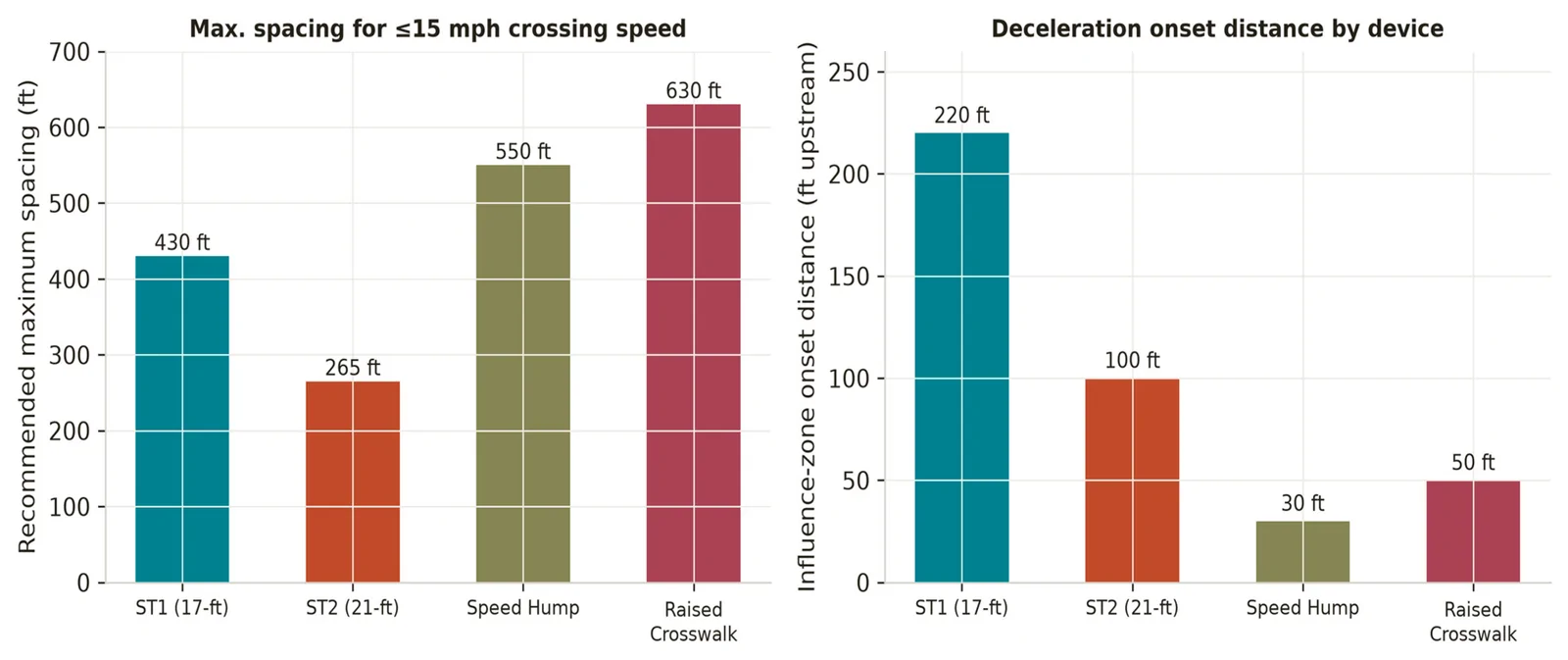
(**Left**): kinematically derived maximum recommended device spacing to maintain an 85th-percentile downstream crossing speed at or below 15 mph. (**Right**): upstream influence-zone onset distance by device.

Relative to the corridor’s base free-flow capacity input of 1180 vphpl at v/c = 1 (30 mph free-flow speed), the reconstructed device capacities correspond to reductions of 20–33% depending on device and spacing (Table 12, Figure 7). Capacity reduction increases monotonically as spacing decreases at every device for which multiple spacings are available, confirming the expected overlap effect between adjacent devices’ deceleration–recovery envelopes. The speed hump shows the largest reduction at the tightest spacing evaluated (33% at 350 ft), while Speed Table 2 shows the smallest reduction at the widest spacing evaluated (20% at 1050 ft). Combining the kinematic recovery-zone lengths from Table 6 with VISSIM-simulated downstream crossing speeds, Figure 8 (left) shows the kinematically derived maximum spacing at which the 85th-percentile downstream crossing speed remains at or below the 15 mph pedestrian-safety threshold: 430 ft for Speed Table 1, 265 ft for Speed Table 2, 550 ft for the speed hump, and 630 ft for the raised crosswalk. Figure 8 (right) reports the influence-zone onset distance—the upstream distance at which vehicles first begin measurable deceleration—which is largest for Speed Table 1 (220 ft) and smallest for the speed hump (30 ft), broadly consistent with the wider top-width devices producing an earlier, more gradual approach response.

### 4.7. Statistical Significance of Spacing Effects

One-way ANOVA confirms that mean simulated capacity differs significantly across the three device spacings evaluated (*p* = 0.0001). A Fisher LSD post hoc comparison between the two most operationally relevant spacings, 350 ft and 700 ft, confirms this difference remains significant after pairwise correction (*p* = 0.001), supporting the practical conclusion that spacing decisions in the 350–700 ft range materially affect corridor capacity and are not attributable to simulation noise.

## 5. Discussion

The results demonstrate that a modest camera–GPS sensor-fusion deployment—a single portable video counter per approach and ten instrumented probe vehicles—is sufficient to close the loop from field measurement, through microsimulation calibration, to macroscopic capacity reconstruction for a multi-device traffic-calming corridor. Three findings merit closer discussion. First, the divergence in deceleration behavior between the two speed tables is notable given their geometric similarity. Speed Table 2 (21-ft top width) is associated with both a higher point-estimate mean deceleration (4.52 vs. 3.44 ft/s^2^, Table 4) and a higher zone-average deceleration over a shorter approach zone (1.89 ft/s^2^ over 364 ft vs. 1.38 ft/s^2^ over 537 ft, Table 5) than the narrower Speed Table 1. Because top width alone would be expected to produce a gentler, not sharper, deceleration profile for the wider device, this pattern more likely reflects differences in upstream sight distance, advance signage, or driver expectancy at the two locations than a pure geometric effect—an interpretation that a purely aggregate before/after speed study, without the kinematic trajectory reconstruction enabled by continuous GPS sensing, could not have distinguished.

Second, the sub-1% mean-speed calibration accuracy achieved at every device (Table 6) is markedly tighter than the 5–10% tolerances typically cited as acceptable in the microsimulation calibration literature [25,39], and appreciably tighter than what is achievable when car-following parameters are seeded only from generic literature defaults, as in prior device-spacing microsimulation studies [7,8]. This suggests that fusing dense, distance-referenced GPS trajectory data directly into the calibration objective—rather than calibrating only to aggregate volume or spot-speed counts—can materially improve simulation fidelity at the individual-device level. The asymmetry between the northbound (5.7%) and southbound (12.1%) travel-time validation errors (Table 8), while both are within the accepted 15% threshold, is worth noting: it may reflect direction-dependent driver behavior (e.g., differential sensitivity to the after-hours gate or on-street parking encountered only in one direction) or a residual asymmetry in the calibrated car-following parameters that a single aggregate mean-speed calibration target does not fully resolve. Because both directional errors remain below the 15% FHWA acceptance threshold [39] and every other validation metric in this study (mean-speed calibration, GEH) passes with a substantially larger margin, this asymmetry is judged not to undermine the overall credibility of the calibrated model; nonetheless, a direction-specific (northbound/southbound) car-following calibration, rather than a single corridor-wide target, is recommended in future work to test whether the 12.1% southbound error can be further reduced.

Third, the narrow range of calibrated May–Keller shape parameters (m = 0.550–0.635, Table 10) across four geometrically distinct devices and three spacings suggests that, once the model is anchored to a device- and spacing-specific capacity via the jam-density boundary condition (Equation (7)), the residual shape parameter is relatively insensitive to device type. This is a useful practical finding: it implies that a designer without simulation capacity for a new device could reasonably interpolate a shape parameter from Table 10 and combine it with a spacing-specific capacity estimate (for example, from the kinematic spacing model of Equation (9) or from comparable published capacities) to obtain an approximate speed–flow–density diagram without running a full microsimulation calibration.

The observed relationship between recovery-zone length and recommended maximum spacing (Table 5 and Table 11) also has direct design relevance. Speed Table 2, which exhibited the longest recovery zone (434 ft) among the two speed tables, correspondingly received the tightest recommended maximum spacing (265 ft) to maintain the 15 mph downstream-crossing-speed criterion, while Speed Table 1’s shorter recovery zone (239 ft) supported a substantially wider recommended spacing (430 ft). This inverse relationship between recovery-zone length and safe spacing is intuitive but, to the authors’ knowledge, has not previously been quantified from field-observed kinematic trajectory data at the individual-device level.

Finally, the sensor-fusion architecture demonstrated here is directly extensible toward the “smart” or adaptive traffic-calming concepts recently proposed in the sensors literature [31,32], in which a device’s physical profile (e.g., hump height or crosswalk illumination) is modulated in real time based on an approaching vehicle’s sensed speed and classification. The kinematic feature-extraction model, applied to a real-time GPS or camera-derived vehicle trajectory rather than a post-processed one, could in principle serve as the perception layer for such a closed-loop, sensor-actuated calming system, an application the authors identify as a promising direction for future work.

## 6. Limitations

Several limitations should be considered when interpreting and generalizing these results. First, the study is based on a single four-device corridor in one city; the specific numerical relationships reported here (deceleration rates, capacity reductions, recommended spacings) should not be extrapolated to other roadway contexts, device designs, or driver populations without independent field validation. Second, the underlying GPS trajectory sample is small—ten drivers completing 40 round trips, of which 30 were retained after quality control—which limits the statistical power of any device-to-device comparison of individual trajectory features (Table 5) and means the reported kinematic values should be treated as indicative rather than definitive point estimates. Third, the parametric log–logistic desired-speed distribution (Table 3) was fitted only for the two speed-table devices, because the source field study tabulated a complete seven-point percentile distribution only for those two devices; no equivalent continuous distribution is available for the speed hump or raised crosswalk, limiting the generality of Figure 1’s conclusions to speed-table-type devices. Fourth, the macroscopic capacity model was calibrated using two physically motivated boundary conditions—a fixed jam density and a fixed density exponent—rather than a full nonlinear regression against directly measured density–flow pairs, because the underlying field study did not directly measure traffic density. While this anchoring approach ensures the calibrated curve exactly reproduces the VISSIM-simulated capacity at each device and spacing, the shape of the reconstructed speed–density curve away from the capacity point (Figure 5) is model-derived rather than independently field-validated. Fifth, the spatial kinematic transform a(x) = v·dv/dx (Equation (2)) was adopted instead of a time-based finite-difference acceleration estimate specifically because the field and simulation-exported trajectories are irregularly spaced in time but continuously indexed in position; a direct quantitative comparison against a time-based extraction was not performed in this study, and future work should benchmark the two formulations against a common high-frequency, fixed-time-step trajectory dataset to formally quantify their relative noise sensitivity.

## 7. Conclusions

This study demonstrated a reproducible camera–GPS sensor-fusion workflow for characterizing the safety–capacity trade-off of a four-device traffic-calming corridor, combining a portable video-based volume sensor with survey-grade GPS probe-vehicle trajectories. A distance-referenced kinematic feature-extraction model isolated device-specific approach-deceleration and recovery-acceleration zones directly from field trajectories, while a log–logistic cumulative distribution function provided a compact parametric representation of the field-observed desired-speed distribution (fit RMSE below 0.043 at both speed-table devices). The fused sensor data were used to calibrate a PTV VISSIM microsimulation replica of the corridor to a mean-speed accuracy of 0.71% or better and a GEH statistic below 1.5 at every analysis movement, with independent travel-time validation confirming errors of 5.7–12.1%, both within accepted tolerances. Propagating the validated simulation into a device- and spacing-specific calibration of the May–Keller macroscopic speed–density model reconstructed capacities of 650–775 vphpl at 350–1050-ft device spacing, corresponding to capacity reductions of 20–33% relative to free-flow conditions, and yielded kinematically grounded maximum recommended spacings of 265–630 ft to maintain a 15-mph pedestrian-safety crossing-speed threshold.

These results illustrate the value of continuous, sensor-fused trajectory data—beyond conventional spot-speed or before/after volume studies—for both diagnosing device-specific behavioral responses and quantitatively informing traffic-calming corridor design. Future work should extend the sensor-fusion protocol demonstrated here to a larger, multi-corridor sample to test the generality of the observed deceleration–recovery and capacity-reduction relationships, incorporate direct-density measurement to relax the boundary-condition-based macroscopic model calibration used in this study, and explore real-time application of the kinematic feature-extraction model as the perception layer for emerging sensor-actuated, adaptive traffic-calming devices [31,32]. A further promising direction is the use of artificial-intelligence-based anomaly detection on continuous sensor-fused trajectory streams to automatically flag when driver behavior at a calming device crosses established thresholds—for example, an abnormally high approach speed, an incomplete deceleration, or an erratic recovery acceleration—that are precursors to localized congestion or safety degradation, extending the CNN–LSTM and related trajectory-learning approaches referenced in Section 2.3 [30] from post hoc analysis toward real-time corridor monitoring.

## Figures and Tables

**Table 1:** Novelty comparison with prior traffic-calming evaluation and microsimulation studies.

Study	Field Sensing Basis	Car-Following Calibration	Trajectory-Level Kinematics	Macroscopic Capacity Model	Spacing Guidance
Ewing [4]; Ewing and Kooshian [5]	Aggregate before/after spot speeds	Not applicable (no simulation)	None (point speed only)	None	None (qualitative only)
Lee et al. [6]	Speed, volume, safety indices	Not applicable (no simulation)	None	None	Evaluation index, not a spacing model
García et al. [7]	None (simulation only)	Generic literature defaults	None	Simulation-only capacity output	Spacing scenarios tested, not field-derived
Shirmohammadi et al. [8]	None (simulation only)	Generic literature defaults	None	Simulation-only capacity output	Spacing scenarios tested, not field-derived
This study	Fused Miovision camera + GPS probe-vehicle trajectories	Field-calibrated to GPS mean speeds (Equation (4)), GEH- and travel-time-validated	Distance-referenced approach-deceleration/recovery-acceleration decomposition (Equations (1) and (2))	May–Keller model anchored to VISSIM capacity per device/spacing (Equations (7) and (8))	Kinematically derived, device-specific maximum spacing at 15-mph threshold (Equation (9))

**Table 2:** Study site and sensor specifications.

Parameter	Value
Site	Oakhill Valley Lane, Nashville, TN, USA
Corridor length	5250 ft
Roadway class	Two-lane residential collector, stop-controlled at both ends
Calming devices	Speed Table 1 (17-ft), Speed Table 2 (21-ft), Speed Hump, Raised Crosswalk
Posted/device-zone speed	30 mph/15 mph
Volume sensor	Miovision Scout, 12-h weekday counts (07:00–18:00), per-lane classification
GPS sensor type	WAAS/EGNOS-augmented GPS receiver
GPS horizontal accuracy	5 m (3-D RMS)
GPS velocity accuracy	0.1 m/s
GPS sampling rate	1 Hz
Probe vehicles/drivers	10
Round trips collected/retained	40/30
Microsimulation platform	PTV VISSIM (Wiedemann 74 car-following)

**Table 3:** Field-observed desired-speed percentiles.

Percentile	Speed Table 1 (17-ft), mph	Speed Table 2 (21-ft), mph
First	7.78	9.81
Seventh	8.89	9.82
Fifteenth	9.98	9.93
Fiftieth	13.27	14.20
Eighty-fifth	20.78	21.02
Ninety-fifth	25.03	24.84
One-hundredth	25.92	25.25

**Table 4:** Log–logistic desired-speed distribution fit parameters.

Device	Loc	Scale	Shape	Fit RMSE (Cum. Fraction)
Speed Table 1 (17 ft)	5.605	7.715	3.066	0.0240
Speed Table 2 (21 ft)	5.168	9.082	3.721	0.0429

**Table 5:** Descriptive speed and deceleration statistics by device.

Device	Mean Speed (mph)	SD Speed (mph)	Mean Decel. (ft/s^2^)	SD Decel. (ft/s^2^)
Speed Table 1 (17 ft)	14.66	5.24	3.44	1.08
Speed Table 2 (21 ft)	15.42	4.61	4.52	0.97
Speed Hump	13.60	4.12	2.43	4.12
Raised Crosswalk	14.00	5.00	1.28	0.68

**Table 6:** Kinematic feature-extraction results (zone-average, Equation (2)).

Device	Min. Crossing Speed (mph)	Approach Zone (ft)	Mean Decel. (ft/s^2^)	Recovery Zone (ft)	Mean Recov. Accel. (ft/s^2^)	n Points
Speed Table 1 (17 ft)	14.72	537.0	1.38	239.3	3.02	32
Speed Table 2 (21 ft)	14.80	363.7	1.89	433.7	1.41	19
Raised Crosswalk	14.29	209.7	1.45	94.5	4.80	7

**Table 7:** Speed calibration accuracy by device.

Device	Observed Mean Speed (mph)	Simulated Mean Speed (mph)	Percent Difference
Speed Table 1	14.66	14.64	0.14%
Speed Table 2	15.42	15.39	0.19%
Speed Hump	13.60	13.55	0.37%
Raised Crosswalk	14.00	13.90	0.71%

**Table 8:** Volume (GEH) validation by turning movement.

Turning Movement	Observed Volume (vph)	Simulated Volume (vph)	GEH
Oak Hill School → Robertson Rd and Van Lee Dr	36	28	1.41
Oak Hill School → Churchwood Dr	72	85	1.47
Robertson Rd and Van Lee Dr → Oak Hill School	75	82	0.79
Churchwood Dr → Oak Hill School	48	49	0.14

**Table 9:** Independent travel-time validation (threshold = 15% error).

Direction	Mean Travel Time (s)	Mean Speed (mph)	Percent Error
NB	128.94	19.4	5.7%
SB	127.82	19.6	12.1%

**Table 10:** Simulated capacity (vphpl) by device and device spacing.

Device	350 ft	700 ft	1050 ft
Speed Table 1 (17 ft)	660	690	750
Speed Table 2 (21 ft)	690	740	775
Speed Hump	650	680	740
Raised Crosswalk	670	675	730

**Table 11:** Calibrated May–Keller shape parameter (m) and reconstructed capacity by device and spacing.

Device	Spacing (ft)	Calibrated (m)	Reconstructed Capacity (vphpl)
Speed Table 1 (17 ft)	350	0.628	660
Speed Table 1 (17 ft)	700	0.608	690
Speed Table 1 (17 ft)	1050	0.567	750
Speed Table 2 (21 ft)	350	0.608	690
Speed Table 2 (21 ft)	700	0.574	740
Speed Table 2 (21 ft)	1050	0.550	775
Speed Hump	350	0.635	650
Speed Hump	700	0.615	680
Speed Hump	1050	0.574	740
Raised Crosswalk	350	0.622	670
Raised Crosswalk	700	0.618	675
Raised Crosswalk	1050	0.581	730

**Table 12:** Capacity reduction (%) relative to free-flow base capacity, by device and spacing.

Device	350 ft	700 ft	1050 ft
Speed Table 1 (17 ft)	32%	29%	23%s
Speed Table 2 (21 ft)	—	24%	20%
Speed Hump	33%	30%	—
Raised Crosswalk	30%	25%	—

## Data Availability

The original contributions presented in this study are included in the article. Further inquiries can be directed to the corresponding author.
